# CAP-miRSeq: a comprehensive analysis pipeline for microRNA sequencing data

**DOI:** 10.1186/1471-2164-15-423

**Published:** 2014-06-03

**Authors:** Zhifu Sun, Jared Evans, Aditya Bhagwate, Sumit Middha, Matthew Bockol, Huihuang Yan, Jean-Pierre Kocher

**Affiliations:** Division of Biomedical Statistics and Informatics, Department of Health Sciences Research, Mayo Clinic, 200 First St SW, Rochester, MN 55905 USA

**Keywords:** miRNA sequencing, Analysis pipeline, Differential expression, Variant detection

## Abstract

**Background:**

miRNAs play a key role in normal physiology and various diseases. miRNA profiling through next generation sequencing (miRNA-seq) has become the main platform for biological research and biomarker discovery. However, analyzing miRNA sequencing data is challenging as it needs significant amount of computational resources and bioinformatics expertise. Several web based analytical tools have been developed but they are limited to processing one or a pair of samples at time and are not suitable for a large scale study. Lack of flexibility and reliability of these web applications are also common issues.

**Results:**

We developed a Comprehensive Analysis Pipeline for microRNA Sequencing data (CAP-miRSeq) that integrates read pre-processing, alignment, mature/precursor/novel miRNA detection and quantification, data visualization, variant detection in miRNA coding region, and more flexible differential expression analysis between experimental conditions. According to computational infrastructure, users can install the package locally or deploy it in Amazon Cloud to run samples sequentially or in parallel for a large number of samples for speedy analyses. In either case, summary and expression reports for all samples are generated for easier quality assessment and downstream analyses. Using well characterized data, we demonstrated the pipeline’s superior performances, flexibility, and practical use in research and biomarker discovery.

**Conclusions:**

CAP-miRSeq is a powerful and flexible tool for users to process and analyze miRNA-seq data scalable from a few to hundreds of samples. The results are presented in the convenient way for investigators or analysts to conduct further investigation and discovery.

**Electronic supplementary material:**

The online version of this article (doi:10.1186/1471-2164-15-423) contains supplementary material, which is available to authorized users.

## Background

miRNAs are small non-coding RNAs that regulate mRNAs at the post-transcriptional level by either degrading or blocking its translation and thus affecting protein translation. Changed miRNA expression patterns can be used for diagnostic and prognostic biomarkers [1]. Hybridization based microarray technology has been used for miRNA profiling; however, this technology is hindered by its narrow detection range (low sensitivity for low and saturation for high expressed miRNA), higher susceptibility to technical variation [2], and lack of ability to detect novel miRNAs and structural sequence changes. miRNA profiling through next generation sequencing (miRNA-Seq) overcomes the limitations and has become increasingly popular in biomedical research. However, miRNA-Seq has caused many analytical challenges to researchers, as it needs significant computational resources and bioinformatics expertise. Several tools have been developed over the past few years. mirTools [3] is a web tool that can detect small RNAs and conduct differential expression for a pair of sample. miRNAkey [4] and miRDeep* [5] create a Java interface that allow users to run data locally by dragging and clicking but limit to one or a couple of samples at time. wapRNA [6] can conduct both RNA and miRNA-seq analysis for a single sample through their web server. omiRas [7] is another recent web application for users to upload multiple raw sequence data with differential expression analysis by DESeq [8] between two sample groups.

The common issues with the web-based tools are lack of flexibility (parameter options, outdated reference genome or miRNA annotations), reliability (server down or not functional at all), and control of sensitive patient data. Most of these tools can only process one sample at time or have a data upload limit or require pre-processed data beforehand as input. These constraints significantly limit the use of these existing applications for projects with many samples and complex study designs. None of the tools detect single nucleotide variants (SNVs)/mutations in the coding region of miRNAs, which is increasingly important as it may affect miRNA binding on multiple targets [9–11].

To address these limitations, we have developed a CAP-miRSeq, a comprehensive analysis pipeline for deep microRNA sequencing data, which integrates read pre-processing, alignment, mature/precursor/novel miRNA qualification and prediction, SNV detection in the coding region of miRNA, data visualization, and differential expression between experimental conditions with biological replicates. The results are in a convenient matrix format (both raw and normalized expression count from mature and novel miRNAs) for all samples in a run or project for further analyses. The pipeline is implemented in the Linux environment to run multiple samples in parallel or sequentially through either local installation or Amazon Cloud but can also be run in a single machine mode using a virtual machine for a limited number of samples. Using well characterized data, we demonstrated the pipeline’s superior performances, flexibility, and practical use in research and biomarker discovery.

## Implementation

### CAP-miRSeq components and functions

**Figure 1:**
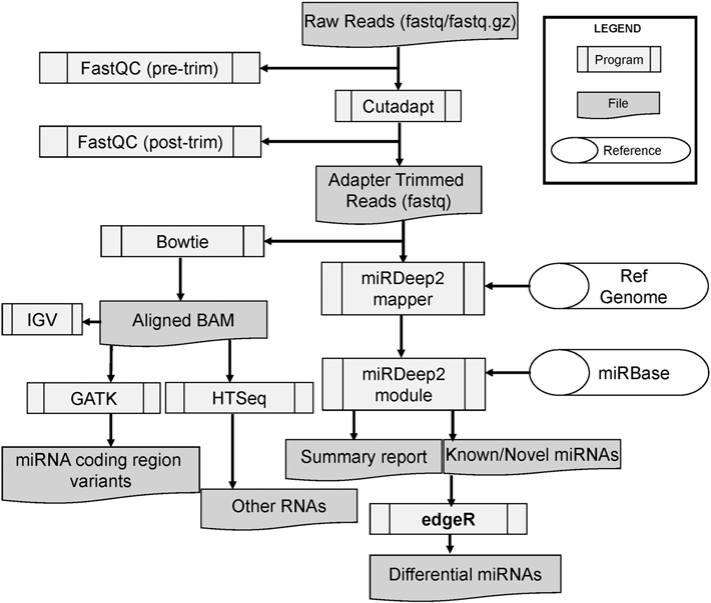
**Workflow diagram of CAP-miRSeq.**

CAP-miRSeq has following major components, each performing a particular function (Figure 1).

A. Read quality assessment and pre-processing: As miRNAs are short (around 22 bps) and the routine sequencing generally has a read length of 50bps or above, this is a critical step for miRNA sequencing data analysis. Reads are first quality checked and low quality bases are trimmed from the 3′ end. Subsequently reads are dynamically trimmed for an adapter sequence by “cutadapt” [12]. Reads less than 17 bases after trimming (by default) are discarded. Second quality check is performed after the trimming to evaluate the read length distribution which is expected to be centered at 22 bases for a good miRNA-seq library preparation.
B. Alignment: The pipeline conducts two alignment processes for trimmed reads, one used internally for miRDeep2 [13] to quantify and predict novel miRNAs and another for all RNA quantification, data visualization and miRNA variant detection, both using the popular alignment tool Bowtie [14]. The miRDeep2 mapper module converts fastq reads to fasta where unique sequences are counted for alignment. The second alignment generates the standard bam which can be used for RNA quantification and variant detection.
C. miRNA prediction and quantification: This process is handled by miRDeep2 as it not only quantifies reads mapped to miRNA coordinates but also evaluates the miRNA compatibility of the sequence where reads are stacked, i.e., whether it can form a hairpin structure of a pre-miRNA and the read distribution at different part of the structure (5′, 3′ mature miRNA, loop) follows the pattern of Dicer processing [13, 15]. Novel miRNAs are identified in a similar manner for the genomic regions not defined by miRBase annotation. A confidence score of a true miRNA is assigned to each miRNA detected.
D. All captured RNA quantification: miRNA-seq library may contain a variety of transcripts. By quantifying all RNAs and their percentages in the library, we can evaluate the quality of the miRNA-seq experiment and utilize the information for other captured small RNAs. CAP-miRSeq quantifies all RNAs as defined in the latest GENCODE annotations (release 18) and displays the percentage of each RNA category in a pie chart for QC purpose.
E. SNV detection in the coding region of known miRNAs: The aligned bam file is processed using GATK [16] to call SNVs in miRNA primary transcripts. If a SNV is located in the seed region of the mature miRNA (1–8 base of 5′ end), it is flagged in the variant report.
F. Sequence data visualization: CAP-miRSeq has two ways of visualization. For each miRNA, known or predicted, a PDF file is generated for its hairpin structure, along with aligned reads at each portion of hairpin structure. An xml configuration file is generated automatically for IGV (http://www.broadinstitute.org/igv/) for users to visualize aligned reads and SNVs.
G. Data reports: CAP-miRSeq generates several reports. The first is a high level summary for each sample’s alignment statistics and number of miRNAs detected. The merged reports of raw count and normalized count in reads-per-million (RPM) for known miRNAs of all samples in matrix format make it easier for further analyses. A URL link to miRBase is provided for each miRNA for detailed annoations. As predicted novel miRNAs only have genomic coordinates and can differ from sample to sample, it would be difficult to conduct comparison for a large number of samples. On the other hand, a true novel miRNA is often detected in multiple samples. We have implemented a strategy to merge a commonly detected novel miRNA across samples if their start/end coordinates overlap by at least 80%. A new genomic coordinate is created for these miRNAs using the outer most coordinate. We have observed that most commonly detected miRNAs have the same or very similar coordinates, which further verify a true novel miRNA.
H. Differentially expressed miRNAs between biological conditions: One of the main motivations behind miRNA profiling is the identification of differentially expressed miRNAs between two experimental conditions. The CAP-miRSeq implements edgeR, empirical analysis of digital gene expression data, from Bioconductor (http://www.bioconductor.org/) described previously [17]. The model uses empirical Bayes estimation and exact tests based on the negative binomial distribution. The analysis can be conducted between two groups, either paired or non-paired samples. Differential p value distribution and volcano plot are provided to visualize the magnitude of the differences between the compared conditions.
I. Pipeline implementation: The pipeline is implemented with combination of shell, perl, python, and R scripts in a Linux environment. It can be run sequentially on a single machine or in parallel in a cluster with Sun Grid Engine (SGE). The package can be installed locally with a set-up script and detailed instructions. For users not comfortable with the installation, we provides a virtue machine image of the software and users can load it into their virtue machine player such as Oracle VM VirtualBox (https://www.virtualbox.org/) to use the software directly for a small scale study. An Amazon Machine Image is also provided for users to take an advantage of the powerful computational environment.

### Test datasets

1. MCF7 cell line: This dataset has 4 miRNA sequencing libraries from MCF7 breast cancer cell line as described previously [13] (Accession number: GSE31069). Two libraries are control and 2 are after Dicer knock-down. For the control and experiment samples one was isolated from cytoplasmic fraction and the other from all cell content. The data was generated from Illumina Genome Analyzer II at 36 bps. Further details are summarized in Table 1. This unique dataset is used to demonstrate: (a) the multiple sample processing by parallel computing; (b) merged data report and summary; (c) differential miRNA expression before and after Dicer knock-down through paired design and consideration of normalization when majority of miRNAs are reduced from the Dicer knock-down; (d) The ability of CAP-miRSeq in discerning the Dicer effect on blocking miRNA biogenesis.

**Table 1:** **Data summary for MCF7 miRNA-seq data**

SRA_ID	Treatment	miRNA source	Read number
SRR326279	control	cytoplasm	15,493,265
SRR326280	control	total	14,670,735
SRR326281	Dicer knock-down	cytoplasm	9,237,490
SRR326282	Dicer knock-down	total	8,689,337
2. Clear cell renal cell carcinoma (ccRCC): This dataset contains 10 pairs of tumor and normal kidney for patients with renal cell carcinoma [18] (GEO accession#: GSE24457). miRNA-seq was conducted by Illumina sequencer. In the study, several up (miR-210, miR-122, miR-155, and miR-224) and down (miR-184 and miR-206, miR-200c, miR-141, miR-200a, miR-200b, and miR-429) expressed miRNAs in cancer relative to paired normal kidney were identified and validated through RT-PCR previously [18]. Notably, the study identified a cluster of miRNAs in chromosome Xq27.3 that were all down expressed (miR-506, miR508-3p, miR-509-5p, miR-509-3p, miR-509-3-5p, miR-510 and miR-514) as a feature of the cancer and further validated by RT-PCR. We used the dataset to test our pipeline whether the same results could be replicated.

## Results

### Pipeline performance

CAP-miRSeq is mainly developed for a cluster environment to parallelize multiple jobs for faster processing so the run time is roughly the time needed for a single sample to complete the whole pipeline, plus the time such as to merge multiple samples and create summary reports. When all 4 MCF7 libraries were run simultaneously in our cluster environment, it took about 4–5 hours to complete with maximum 10 G memory usage. The sample SRR326279 has the highest number of reads and when it was run through the interactive mode, it took 5 hours for the whole process with 4G memory usage.

### Representative outputs from the core module of CAP-miRSeq

**Figure 2:**
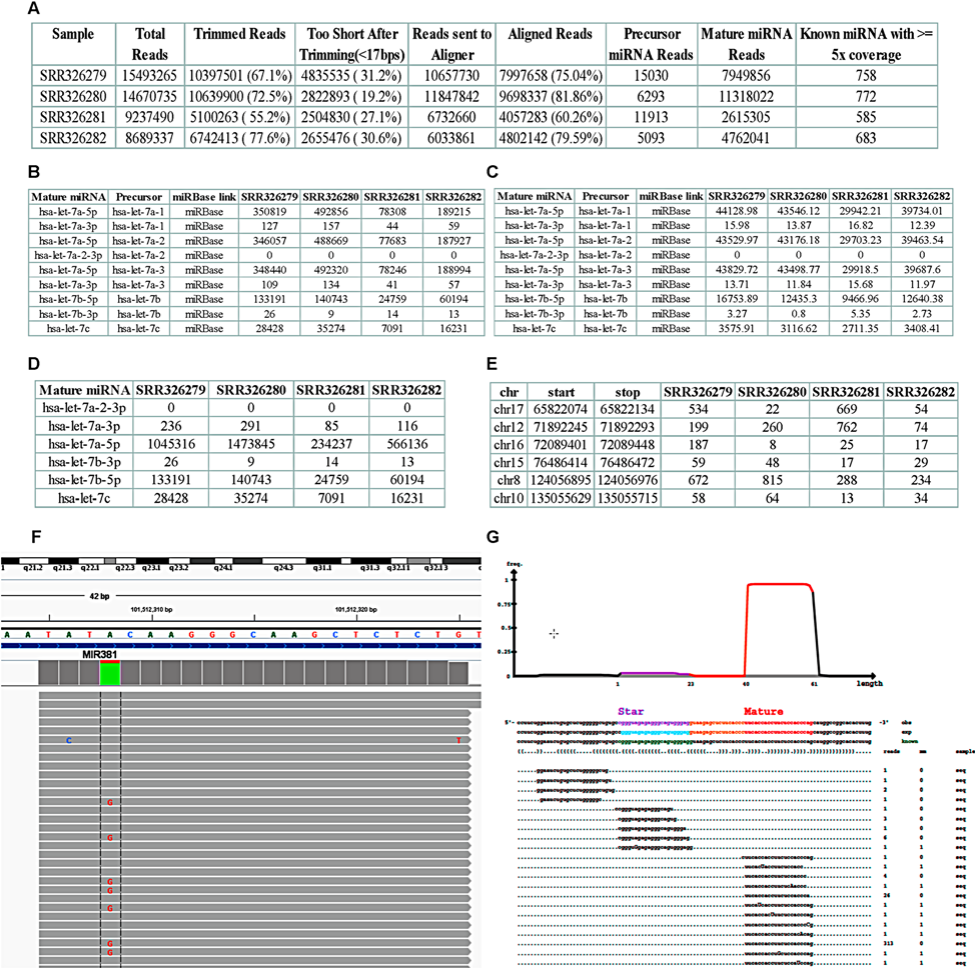
**Example outputs from CPAP-miRSeq (MCF7 dataset). A**. Summary statistics of each miRNA-seq library processed. **B**. Raw mature miRNA expression table, weighted count for miRNAs coded by multiple pre-miRNAs. **C**. Normalized mature miRNA expression table by number of reads per million (RPM). **D**. Summarized mature miRNA expression table for those with multiple pre-cursors. These miRNAs are indistinguishable by sequence but have the same biological effect. This table is used for final differential expression analysis. **E**. Merged novel miRNA expression across samples. **F**. Integrative Genome Viewer (IGV) to visualize sequence level data for a single nucleotide variant. **G**. Mapped read distribution in different parts of the hairpin structure of miRNA precursor which provides a strong evidence of an authentic miRNA.

The first high level report summaries the number of reads from a sequencer, the number of reads got trimmed, the number of reads tossed after trimming because of shorter than specified minimum length, the number of reads aligned to the reference genome, to mature and precursor miRNAs, and the number of miRNAs detected with at least 5 reads each sample (Figure 2A). This will give investigators a quick grasp on how the sequencing experiment performs. Three mature miRNA quantification reports in a matrix format are generated. As a miRNA can be coded by multiple precursors, the weighted miRNA count attached to each miRNA precursor is reported in “miRNA_expression_raw.xls”, with mature and precursor miRNA IDs and hyperlink to miRBase (http://www.mirbase.org/, Figure 2B). The similar but normalized per million of mapped reads to miRNAs is provided in “miRNA_expression_norm.xls” so investigators can compare relative expression quickly among samples (Figure 2C). Noted is that in some cases where majority of miRNAs are reduced due to the global inhibition of miRNA biogenesis such as Dicer knock-down, the commonly used normalization methods are often not appropriate as illustrated later in the manuscript. Additionally, matured miRNAs coded by different precursors with the exact same sequence are impossible to distinguish. To facilitate further analysis, the expression of such miRNAs is summed across different precursors from a weighted raw count report with a unique mature miRNA ID (Figure 2D). Novel miRNAs across samples are merged into a single report (Figure 2E) where original and merged coordinates and a true miRNA confidence score are recorded. An IGV session file is automatically generated for users to load aligned bam files to visualize sequence read data (Figure 2F). The read distribution to each part of precursor miRNA sequence can also be examined for each miRNA (Figure 2G).

### Quantification of all captured RNAs

**Figure 3:**
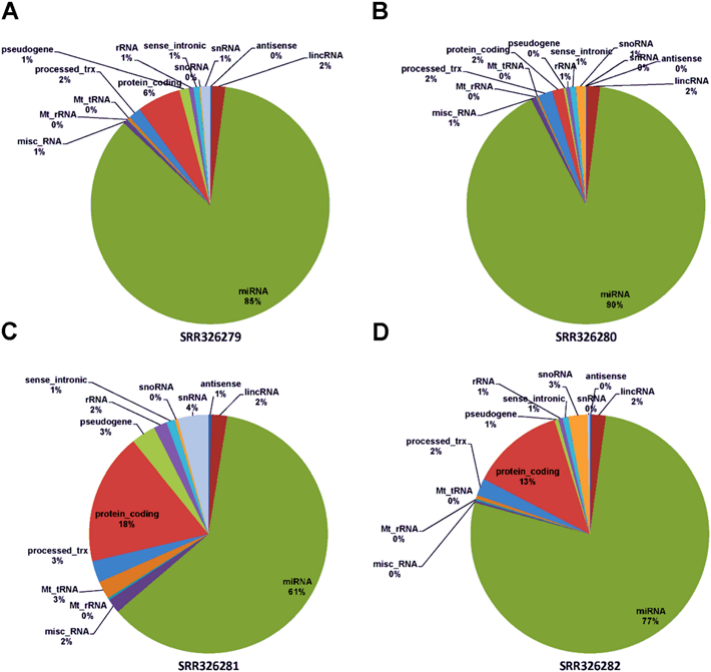
**Distribution of all detected RNAs by category for MCF7 dataset. A**. RNA extracted from cytoplasm without the Dicer treatment. **B**. RNA extracted from total RNA (both cytoplasm and nucleus) without the Dicer treatment. **C**. RNA extracted from cytoplasm with the Dicer treatment. **D**. RNA extracted from total RNA (both cytoplasm and nucleus) with the Dicer treatment. miRNA expression is significantly repressed in the Dicer treated cell lines **(C and D)**.

While the core module discovers miRNAs, it is necessary to quantify all RNAs in the library for quality control and maximize the use of the data. Through intersecting GENCODE annotation for the 4 miRNA sequencing libraries of MCF7, we found that for both cytoplasmic and total miRNAs, the Dicer depleted cells had a significant reduced miRNA proportion (reduced from 85% to 61% and from 90% to 77% for cytoplasm and total RNA, respectively, Figure 3), as the Dicer disrupted the miRNA biogenesis from pre-miRNAs to mature miRNAs. This also confirmed good miRNA-seq library preparation as majority of the RNAs were from miRNAs. A low miRNA percentage often would suggest failed miRNA purification or low miRNA content. The step also quantified many other RNAs such as snoRNA, snRNA, tRNA or lincRNA for interested investigators.

### Differential expression of miRNA before and after Dicer treatment

The MCF7 dataset was used for illustration. The pipeline generated a boxplot of miRNA expression before and after normalization and multidimensional scaling plot (Additional file 1,A and B). Differential p value distribution and volcano plot were also created for overall examination of differential expression magnitude and significance (Additional file 1,C and D). Library size normalization, i.e., using the total number of reads mapped to miRNAs as a normalization factor to standardize different depths of sequencing, is routinely carried out and works well most of time. However, in some special cases where miRNAs are globally reduced such as the blockage of their biogenesis from Dicer knock-down or gene mutations [19], this normalization would artificially boost the expression of reduced miRNAs and obscure true differences. Using the number of reads aligned to genome or the total number of reads generated or a subset of miRNAs that are not affected by miRNA biogenesis is preferred. Indeed, when we used the number of aligned reads to miRNAs as a normalization factor, only slightly more miRNAs were down-expressed after Dicer treatment (Additional file 1D). However, this was largely corrected by using the number of reads aligned to the whole genome as the library size for normalization (Additional file 2). We used this special case to illustrate that it may not be wise to conduct differential expression blindly before making sure that a default normalization method is appropriate. For this reason, we do not recommend running differential expression at the time of sample processing but after the data is fully quality assessed and the study design is fully understood.

### miRNA coding region variant detection

We used the two control cell lines SRR326279 SRR326280 (without Dicer knockdown) with deeper sequencing to detect and compare SNVs for illustration. At the minimum 10X coverage and genotyping quality score greater than 30, 225 SNVs were detected in the coding region of miRNAs in either sample, of which 200 (89%) were confidently detected in both samples. For the remaining 25 positions, all but 2 positions had the same alternative allele but not at sufficient frequency to call a variant in one of the samples. Among the 200 SNVs, 66 were in the mature miRNA and others in the precursor miRNAs. The high concordance between the two replicates demonstrated the variant call reliability.

### Dicer knock-down leads to reduced miRNA expression

After the Dicer treatment, the total miRNAs in MCF7 cell line were reduced 23.87% and 13.40%, respectively for miRNAs extracted from cytoplasm and whole cell component among all RNA transcripts (Figure 3). There was essentially no change for rRNA (increase of 1.16% and 0.02%), snoRNA (increase of 0.23% and 1.36%), and snRNA (2.9% and 0.19%). On the contrary, protein coding mRNAs increased about 11% in both cytoplasm and whole cell component RNAs. Differential miRNA analysis between the Dicer knock-out and controls by paired analysis showed 246 miRNAs with p value less than 0.05, among which 166 (67%) were down and 80 (33%) were up expressed (Additional file 2). The miRNAs that were not repressed were likely matured by Dicer independent pathways [20–22]. These results were consistent with what was previously reported [13].

### Deregulated miRNAs in the dataset of ccRCC

**Figure 4:**
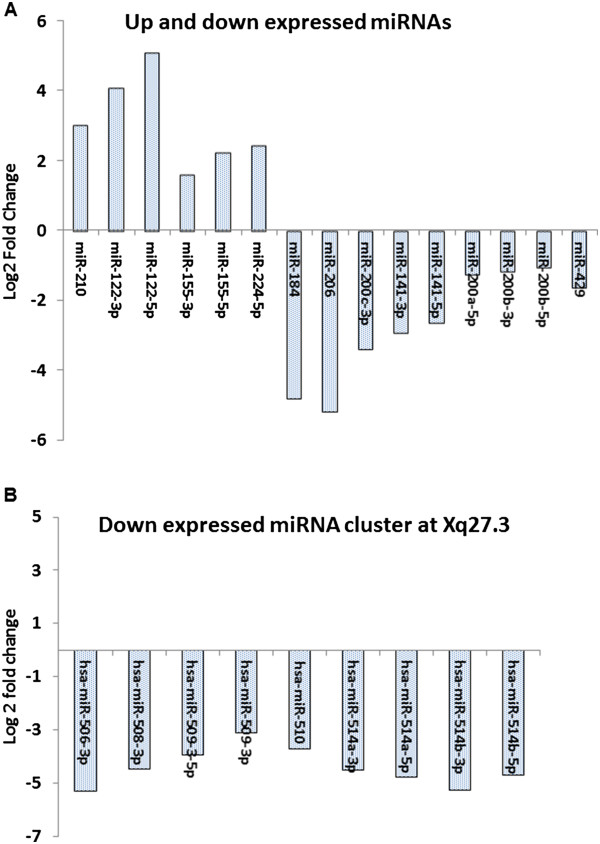
**Changed miRNAs in renal cancer carcinoma validated by CAP-miRSeq. A**. Significantly up and down expressed miRNAs in renal cell carcinoma compared with paired normal kidney as previous reported and described in the text. **B**. Significantly down expressed miRNAs in chromosome Xq27.3 locus as reported.

Using CAP-miRSeq to process the raw fastq data and obtain the mature miRNA expression for 20 samples (10 tumor and normal pairs), we then conducted differential expression between tumor and normal samples by paired edgeR model. The analysis found 144 miRNAs with differential expression p value < 0.01, of which 103 with false discovery rate < 0.05. All 11 changed miRNAs reported in the original report [18] were confirmed to be similarly up or down expressed from our analyses (Figure 4A). Additionally, we observed that all miRNAs in chromosome Xq27.3 locus were significantly down expressed in ccRCC tissues (Figure 4B). The result further validated the reliability of our pipeline and analysis.

### Comparison with other publicly available tools

We used the sample SRR326279 to compare the known miRNA prediction and quantification with several most recent public tools (Table 2). omiRAS detected 739 mature miRNAs with minimum 2 reads while our CAP-miRSeq detected 860 miRNAs. For the common 739 matured miRNAs, the correlation coefficient is almost 1 (0.999). Novoalign miRNA module does not perform miRNA prediction. We quantified the mature miRNAs using bedtools where 881 mature miRNAs had minimum 2 reads. The correlation coefficient with CAP-miRSeq is 0.95. miRTools2 only reported 172 mature miNRAs with 2 reads or above and these miRNAs had overall lower expression compared to CAP-miRSeq, omiRNA or Novalign (with correlation coefficient of 0.81). CAP-miRSeq detected 194 novel miRNAs while mirTools reported 35 novel miRNAs. omiRAS needs at least two samples to run and does not report novel miRNAs for each individual sample.

**Table 2:** **Comparison of different tools in miRNA detection**

	CAP-miRSeq	omiRAS	miRTools2	Novoalign
Version	v1.1	12/2013	v2	2.07.13
Aligner	Bowtie	Bowtie	SOAP	Novoalign^+^
Ref Genome	hg19	hg19	hg19	hg19
Time	5 hrs	12 hrs	Variable	6 hrs
Memory	4 GB	-	-	10 G
Mature miRNA (> = 2)	860	739	172	881
Novel miRNA	194	NA*	35	No prediction
Correlation with other tools	-	0.99	0.81	0.95

*NA–omiRAS requires at least two samples to run with differential expression analysis; however, it does not report novel miRNAs for each individual sample. ^+^Novoalign is just an alignment toot and it does not perform miRNA prediction and quantification. omiRAS and miRTool2 are web based tools and no memory usage information is available.

### Sequence depth and miRNA capture

**Figure 5:**
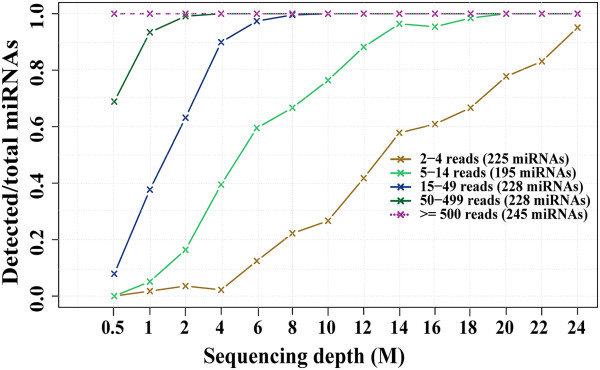
**Simulation result of sequence depth vs. miRNA capture.** The original library has ~25.4 million single end reads. miRNAs are defined as expressed at an arbitrary cutoff of > =2 reads and clustered into five expression level groups based on the number of assigned reads. The original library is randomly sampled at 14 different depths from 0.5M to 24M. For each of the five groups from each sub-library, the number of miRNAs with > =2 reads are calculated and the ratio over the corresponding total from the original library is displayed. About 78% of expressed miRNAs can be detected at sequence depth of 12–18 million of reads.

One of the common questions in miRNA-Seq design is how deep miRNA sequencing needs to be. To provide some guidance to investigators, we conducted a simulation study with a miRNA-Seq sample with 25 million of reads. The experiment started from the complete dataset and then randomly drew 0.5, 1, and 2 to 24 with 2 million increments. At the full data, 1,121 miRNAs were detectable with at least 2 reads and these miRNAs were binned into 5 levels of expression. Almost all miRNAs with expression greater than 15 reads (58% of expressed miRNAs) were detectable at 6–8 million of sequence reads. With 12–18 million of reads, 78% of all expressed miRNAs could be detected. Although further increase of sequence depth could capture very low expressed miRNAs, their biological significance can be questionable thus a sequence depth of at least 10 million reads per sample could get a reasonable capture for expressed miRNAs (Figure 5) assuming the miRNA-Seq library mostly consists of miRNAs.

## Discussion

With high multiplexing and a low number of required reads for a sufficient sequencing depth, miRNA-seq becomes a popular platform for miRNA profiling with tens or even hundreds of samples, which makes the web based applications or applications that process one sample at time impractical. Herein we have presented a powerful and comprehensive analytical pipeline flexible to process many samples simultaneously for users with a cluster environment or sequentially for those who don’t have the computing capacity. The pipeline generates merged reports of known and novel miRNAs for all samples to make further analyses easier. Optionally, the users can request differential expression analysis for grouped or paired design, SNVs or mutation detection in the coding region of miRNAs. Through the well characterized datasets, we have demonstrated its superior performances, reliability and flexibility.

The relative performance of different miRNA-Seq tools was compared comprehensively previously [23]. The sensitivity and specificity of different tools in detecting known or novel miRNAs appear different among different species of data. miRDeep was shown with high specificity in known miRNA detection and high sensitivity in novel miRNA prediction [23]. miRDeep2 [13], the overhaul version of miRDeep, is used in our pipeline for miRNA detection and quantification and demonstrates the similar performances in our comparisons.

Some published tools have the function performing differential miRNA expression analysis between samples [3, 4, 7, 24]. However, miRanalyzer, CPSS and miRNAKey only allow a pair of samples using Chi Square or Fisher’s exact on raw read counts. miRTools2, the updated version of miRTools and omiRas allow users to perform differential expression analysis between two or more samples. However, the former needs each sample processed separately ahead of time while omiRas can not handle paired design. The potential issues with the “automatic” differential expression analysis are that it conducts the analysis before data quality is thoroughly examined, which is the must-step for any genomic data analysis. Secondly, most analysis tools use library size calculated from the mapped reads to miRNAs as a normalization factor, which in some cases is not appropriate as we illustrated where majority of miRNAs are reduced as the result of Dicer knock-down. Although we provide the convenient option to conduct differential expression analysis when running the pipeline, it is strongly recommended to be done after a rigorous quality assessment is completed and the study design is fully understood. A standalone script is provided for the post pipeline differential analysis in our package.

SNVs or mutations in miRNA coding region can have a significant implication because of the miRNAs’ broad binding and action profiles. None of the miRNA-seq tools identify the variants/mutations from miRNAs using a reliable variant caller. We implemented the most commonly used GATK for variant call. From MCF7 cells, we found many high confidence SNVs in the coding regions of miRNAs and some were in the seed region of mature miRNAs. As the functional implications of these variants can not be predicted in the non-coding regions of the genome by current prediction tools and miRNAs often have RNA editing events [25–27], further investigation is needed for their biological implications.

In our comparison with other tools, we have obtained very good correlation with omiRAS and Novoalign miRNA module. The high correlation with omiRAS is not a surprise as it also uses miRDeep as a miRNA prediction tool. The slightly lower correlation with Novoalign is likely due to the fact that Novoalign does not have miRNA prediction step and a detected miRNA is simply the number of aligned reads in the known miRNA annotation. We are not sure why miRTools2 only reported 172 mature miNRAs (about a fourth of other tools) with systematic lower expression from their default settings even though the same reference genome version and miRNA annotation were used. We suspect the parameter of keeping a randomly selected alignment for a read with multiple alignments may contribute to the discrepancy or it might not count the isomiRs.

Other recent tools that were evaluated but not presented include wapRNA, miRDeep*, and CPSS. Both wapRNA and miRDeep* only allow processing one sample at a time and do not report mature miRNA expression (but step-loop region), which is not directly comparable with CAP-miRSeq and others. CPSS did not return any result in spite of several tries.

## Conclusions

CAP-miRSeq is a powerful and flexible tool for users to process and analyze both a small and large number of miRNA-seq samples quickly. The results of both known and novel miRNAs are presented in the merged and convenient format for investigators or analysts to conduct further investigation and discovery. The simultaneously called variants in the coding regions of miRNAs can be used to investigate gene regulation mechanism and phenotype or disease associations.

### Availability and requirements

**Project name:** CAP-miRSeq: a comprehensive analysis pipeline for microRNA sequencing data.

**Project home page:**http://bioinformaticstools.mayo.edu/research/cap-mirseq/.

**Operating system(s):** Linux.

**Programming language:** Perl, Python, R and BASH.

**Other requirements:** Java (7u45), FastQC (0.10.1), Bowtie (0.12.7), Samtools (0.1.19), Bedtools (2.17.0), HT-Seq (0.5.3p9), miRDeep2 (2.0.0.5), VCFTools (0.1.11), GATK (2.7-2-g6bda569), Picard (1.77).

**License:** GNU GPLv2.

**Any restrictions to use by non-academics:** None.

## Electronic supplementary material

Additional file 1: **QC visualization and differentially expressed miRNAs between experimental conditions.** A. boxplot of raw miRNA expression. B. multi-dimentional scaling for the 4 miRNA-seq libraries. Samples are separated by the RNA extraction method in the first principal component (X-axis) and the Dicer treatment in the second principal component (Y-axis). C. histogram of differential expression p value. D. volcano plot of differntially expressed miRNAs (red highlight for those with false discoveray rate less than 0.05). (TIFF 440 KB)

Additional file 2: **2 M-A plot of differentially expressed miRNAs between the Dicer knock-down and controls.** Instead of using the reads aligned to miRNAs, the total number of aligned reads was used as a normalization factor. miRNAs were largely repressed (over 2/3) due to the Dicer inhibition. A small number of miRNAs were unaffected or more expressed (mostly in the low expression range), which may be random noise or go through alternative pathways independent of Dicer. (PNG 73 KB)
