# Supplementary figures and images for: CAP-miRSeq: a comprehensive analysis pipeline for microRNA sequencing data
Source: BMC Genomics. 2014 Jun 3;15(1):423. doi: 10.1186/1471-2164-15-423 (PMC4070549; doi:10.1186/1471-2164-15-423)

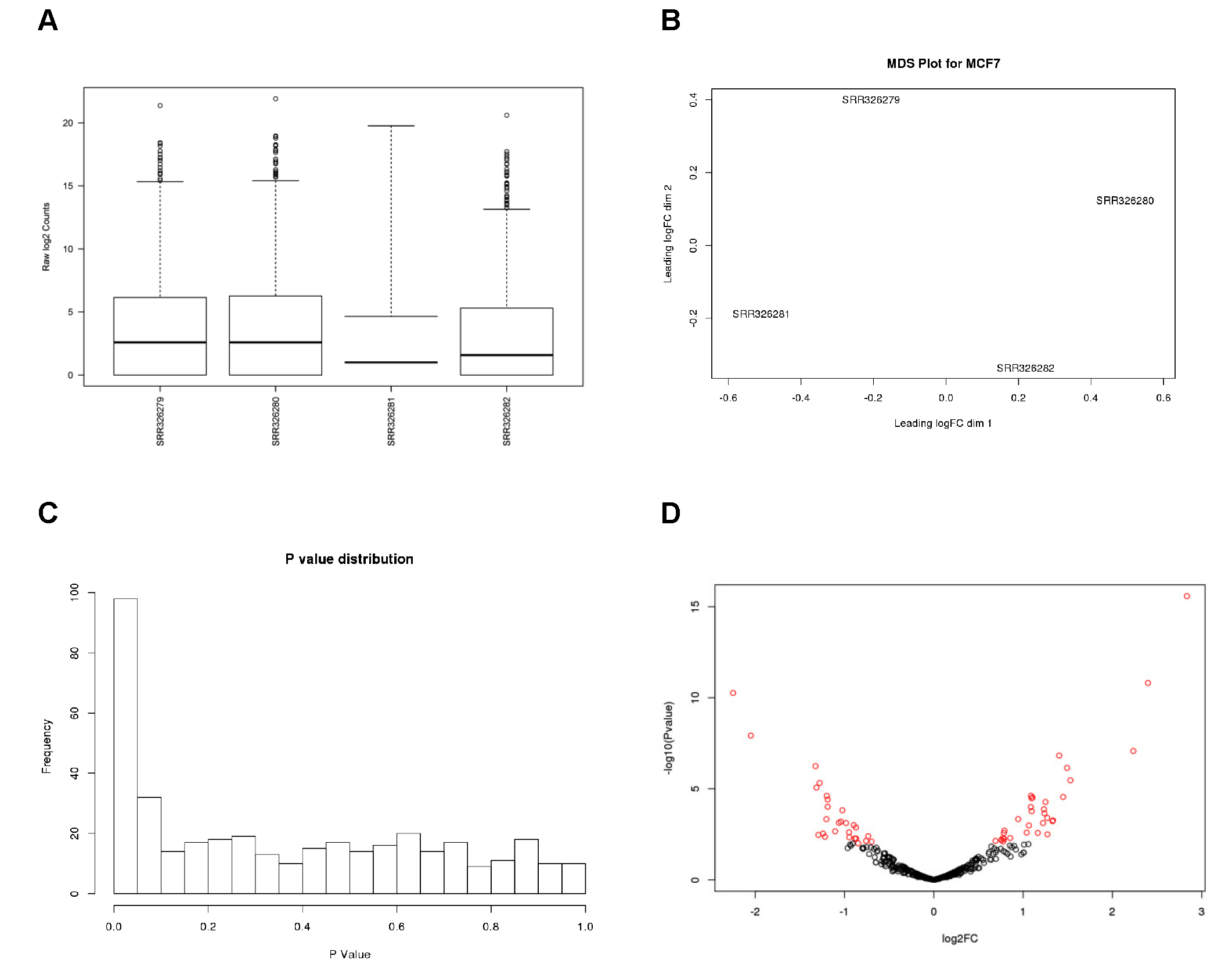

Supplement: Supplementary file 1 — Additional file 1: QC visualization and differentially expressed miRNAs between experimental conditions. A. boxplot of raw miRNA expression. B. multi-dimentional scaling for the 4 miRNA-seq libraries. Samples are separated by the RNA extraction method in the first principal component (X-axis) and the Dicer treatment in the second principal component (Y-axis). C. histogram of differential expression p value. D. volcano plot of differntially expressed miRNAs (red highlight for those with false discoveray rate less than 0.05). (TIFF 440 KB) [file 12864_2014_6123_MOESM1_ESM.tiff]

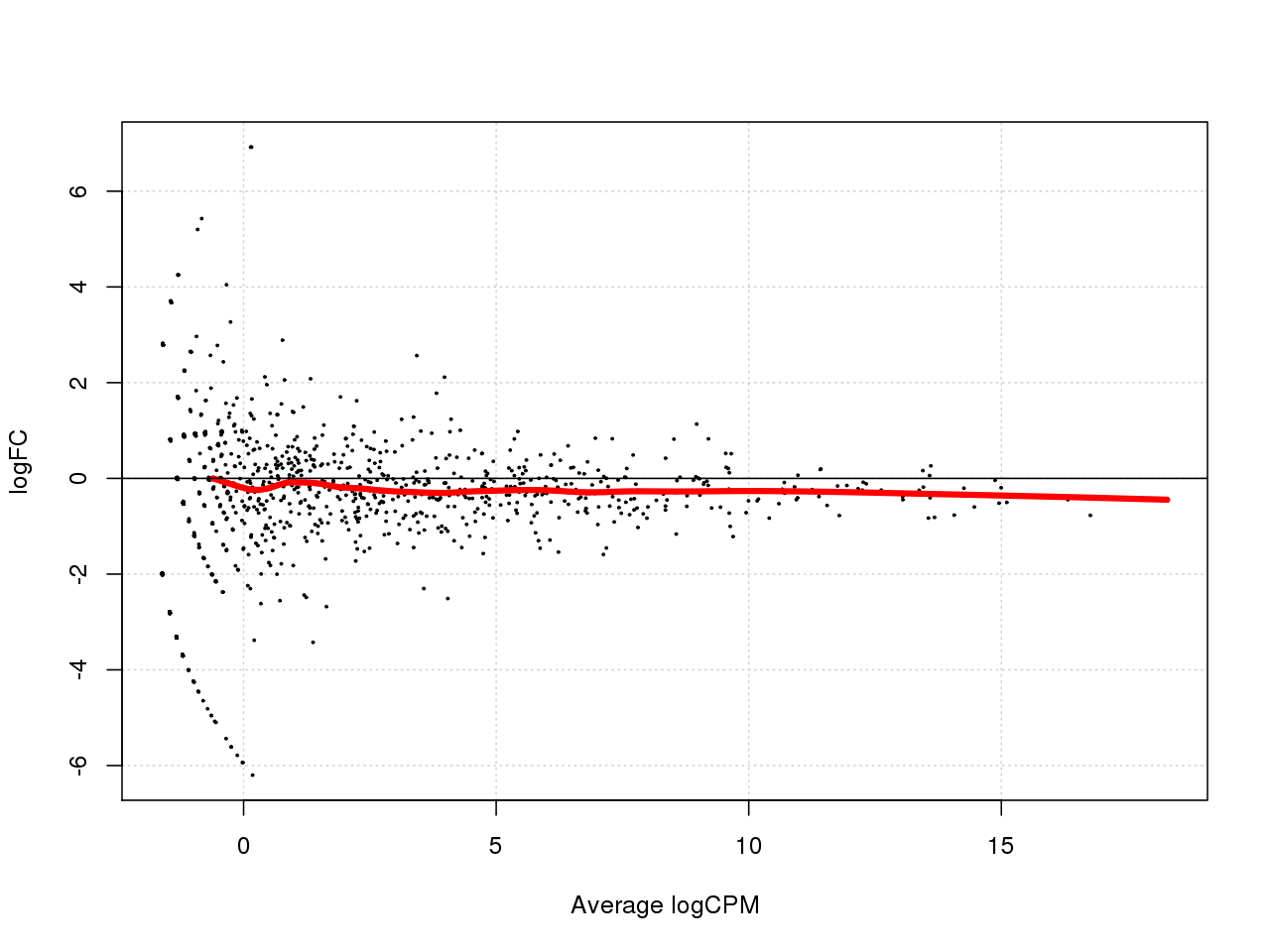

Supplement: Supplementary file 2 — Additional file 2: 2 M-A plot of differentially expressed miRNAs between the Dicer knock-down and controls. Instead of using the reads aligned to miRNAs, the total number of aligned reads was used as a normalization factor. miRNAs were largely repressed (over 2/3) due to the Dicer inhibition. A small number of miRNAs were unaffected or more expressed (mostly in the low expression range), which may be random noise or go through alternative pathways independent of Dicer. (PNG 73 KB) [file 12864_2014_6123_MOESM2_ESM.png]
